# One-Step Synthesis of Environmentally Friendly Superhydrophilic and Superhydrophobic Sponges for Oil/Water Separation

**DOI:** 10.3390/ma12071182

**Published:** 2019-04-11

**Authors:** Yo Seph Lee, Yong Taek Lim, Won San Choi

**Affiliations:** Department of Chemical and Biological Engineering, Hanbat National University, Daejeon 305-719, Korea; yoseph1993@naver.com (Y.S.L.); iyabanju12@naver.com (Y.T.L.)

**Keywords:** Oil/water separation, superhydrophilic, superhydrophobic, environmental remediation

## Abstract

Environmentally friendly superhydrophilic and superhydrophobic sponges were synthesized using a one-step approach for oil/water separation. A superhydrophilic or superhydrophobic sponge (MFS/CC-DKGM or MFS/CC-PDMS) was synthesized by one-step coating of melamine formaldehyde sponge (MFS) with a mixture of calcium carbonate (CC) rods and deacetylized Konjac glucomannan (DKGM) [or polydimethylsiloxane (PDMS)]. The MFS/CC-PDMS showed excellent absorption capacity, which reached 52–76 g/g following immersion into various types of oil/water mixtures. Furthermore, the MFS/CC-DKGM and MFS/CC-PDMS exhibited excellent water- and oil-flux performances, which reached 4,702 L/m^2^ h and 19,591 L/m^2^ h, respectively, when they were used as filters. The MFS/CC-DKGM and MFS/CC-PDMS maintained their wettability characteristics relatively well after the chemical, thermal, and mechanical stability tests.

## 1. Introduction

Since oily wastewater has been increasingly produced over the years by industry and daily life activities, it has become a severe environmental issue and threat. Various materials and techniques for oil/water separation have been extensively reported [1,2,3,4,5,6,7,8,9,10,11,12,13,14]. Superhydrophilic or hydrophilic materials that are partially prewetted with water selectively remove water from oil/water mixtures by filtration or absorption [12,13,14]. For oil removal, oil is normally filtered or absorbed by superhydrophobic or hydrophobic materials [9,10,11]. Since oil/water mixtures produced from industries occasionally contain heavy metals and toxic organic compounds, there is an increasing demand for oil/water separation and pollutant remediation [15,16,17,18,19,20,21,22,23]. Nanomaterials have been widely used as component materials for synthesis of superhydrophilic or superhydrophobic materials because the surface morphology and functionality of the abovementioned materials can be tuned by incorporation of nanomaterials [15,16,17,18,19,20,21,22,23]. Nanocatalyst-loaded oil/water separators decompose toxic organic pollutants in water after oil/water separation is achieved [15,16,17]. Other separators with an adsorption function to adsorb heavy metals or toxic organic compounds in water after the oil/water separation are also reported [18,19,20,21,22,23]. However, unintended leakage of nanomaterials into the environment has been reported recently and represents a significant threat to public health and the environment [24,25]. Most reported superhydrophilic or superhydrophobic materials contain nanomaterials, which are very vulnerable to leakage during the separation process. Ironically, superhydrophilic or superhydrophobic materials containing nanomaterials intended for environmental remediation can, themselves, be hazardous to the environment. By using other materials that are part of nature or nontoxic to nature, synthesizing superhydrophilic and superhydrophobic materials can solve the abovementioned problems. Generally, the synthesis and fabrication of superhydrophilic or superhydrophobic materials are complicated and involve multistep processes, which increase the production costs. Development of a one-step process will decrease the production costs and enhance their application possibilities. To the best of our knowledge, no study has reported synthesis of environmentally friendly superhydrophilic and superhydrophobic materials using a one-step process.

Herein, we report a one-step approach to synthesis of environmentally friendly superhydrophilic and superhydrophobic sponges for oil/water separation. Superhydrophilic and superhydrophobic sponges prewetted with water and oil were able to selectively separate water and oil, respectively. The superhydrophilic and superhydrophobic sponges showed excellent absorption capacities and fluxes. The wettability characteristics of the superhydrophilic and superhydrophobic sponges were robust against chemical, thermal, and mechanical stimuli.

## 2. Materials and Methods 

### 2.1. Materials

The melamine formaldehyde sponge (MFS) was purchased from BASF (Berlin, Germany). Hexane (≥95%), soybean oil, canola oil, and silicon oil were purchased from SK Chemicals (Suwon, Korea), CJ (Seoul, Korea), and Shin-Etsu (Tokyo, Japan). Polydimethylsiloxane (PDMS, Sylgard 184) was purchased from Dow chemical (Michigan, USA). Konjac glucomannan (KGM) was purchased from (Fudongfa, Zhejiang, China). Diesel fuel was purchased from a local gas station. Calcium chloride (CaCl_2_, >97%), sodium carbonate monohydrate (Na_2_CO_3_∙H_2_O, >99.5%), ethanol (C_2_H_5_OH, 96.5%), stearic acid (C_18_H_36_O_2_, >98.5%), methylene blue (MB, 100%), oil red O (100%), and n-hexane (C_6_H_14_, 95%) were purchased from Sigma-Aldrich (Saintlouis, USA). Hydrochloric acid (HCl, 35.0%) and sodium chloride (NaCl, 99%) were purchased from Daejung Chemical (Daejeon, Korea). All chemicals were used without further purification. DI water with a resistance of 18.2 MΩ cm was obtained using a Millipore Simplicity 185 system (Heal force, Beijing, China). 

### 2.2. Preparation of the Hydrophilic CC Rods

First, 0.36 g of calcium chloride (CaCl_2_, >97%) was completely dissolved in 4 mL of deionized (DI) water. Next, 10 mL of ethanol (C_2_H_5_OH, 96.5%) was added dropwise into the calcium chloride solution. Then, 0.35 g of sodium carbonate monohydrate (Na_2_CO_3_·H_2_O, >99.5%) was completely dissolved in 10 mL of DI water in another vial. Four mL of ethanol was added dropwise into the sodium carbonate monohydrate solution. The prepared calcium chloride solution was added dropwise into the sodium carbonate monohydrate solution. The resulted solution was stirred under magnetic stirring for 30 min. The precipitated CC rods were washed three times with DI water and dried in an oven (JSR, Tokyo, Japan) at 50 °C for 4 h.

### 2.3. Preparation of the Hydrophobic CC Rods

First, 0.07 g of stearic acid (C_18_H_36_O_2_, >98.5%) was completely dissolved in 10 mL of ethanol. Then, 0.2 g of CC rods was added to the stearic-acid solution under magnetic stirring for 20 min. The hydrophobic CC rods were washed three times with ethanol and dried in an oven at 50 °C for 4 h.

### 2.4. Preparation of the MFS/CC-DKGM

First, 25 mg of KGM was added to a mixture, containing 10 mL of DI water and 100 mg of CC rods, under magnetic stirring for 5 min. Then, an MFS (2 × 2 × 2 cm^3^) was added to the resulting mixture and heated in an oven at 90 °C for 2 h. The final product was washed 3 times with DI water.

### 2.5. Preparation of the MFS/CC-PDMS

A PDMS solution (m/m) containing cross-linker (1), monomer (10), and xylene (100) was prepared. Then, 100 mg of hydrophobic CC rods was added to 10 mL of PDMS solution under magnetic stirring for 5 min. A MFS (2 × 2 × 2 cm^3^) was added to the mixture and heated in an oven at 100 °C for 1 h. The final product was washed 3 times with ethanol to remove excess PDMS and CC rods.

### 2.6. Oil/Water Separation by the Absorption Process 

For oil/water separation, a piece of the MFS/CC-PDMS (8 cm^3^) sample was dropped into a beaker containing a mixture of 40 mL of oil (hexane, diesel fuel, soybean oil, silicone oil, or canola oil) and 40 mL of water. The oil absorption capacity of the MFS/CC-PDMS was determined by weight measurements. The weights of the MFS/CC-PDMS, before and after oil absorption, were measured as m_1_ and m_2_, respectively. The weight of the absorbed oil was calculated as the difference between m_1_ and m_2_ [(m_2_ − m_1_)/m_1_]. These experiments were repeated 3 times for each measurement, and the average value was used. The water absorption capacity of the MFS/CC-DKGM was also determined using the abovementioned method.

### 2.7. Selective Oil/Water Separation by the Filtration Process

To test the selective filtration ability of the MFS/CC-DKGM and MFS/CC-PDMS (16 cm^3^), a funnel equipped with each sponge filter was prepared. The upper and lower containers of the funnel compressed each sponge (4 × 4 × 1 cm^3^), which had a thickness of 1 cm. The compressed sponge with a thickness of 0.3 cm was used as a filtration membrane. For water removal, the MFS/CC-DKGM filter was prewetted with water. For oil removal, the MFS/CC-PDMS filter was prewetted with hexane. Then, 40 mL of oil was mixed with 40 mL of water and gradually poured into the funnel. The separated water or oil was collected under the funnel. 

### 2.8. Characterization

SEM analyses were carried out using a Hitachi S-4800 instrument (Hitachi, Tokyo, Japan). X-ray diffraction (XRD) (Rigaku, Tokyo, Japan) patterns were obtained on a Rigaku X-ray diffractometer equipped with a Cu Kα source. The UV-vis absorption spectra were recorded on a UV-vis spectrophotometer (Sinco, Seoul, Korea) (Sinco Evolution 201). FT-IR analyses were carried out using a Sinco Nicolet IS5 instrument (Sinco, Seoul, Korea). TGA analyses were performed using a thermogravimetric analyzer (Sinco TGA N-1500) (Sinco, Seoul, Korea) over a temperature range of 25 °C–800 °C at a heating rate of 10 °C/min under air (flow rate, 60 cm^2^/min). Contact angle measurements were carried out using a contact angle meter (SEO Phoenix 300Touch) (Kromtek, Selangor, Malaysia) at ambient temperature; the volume of the probing liquid was 20 μL. 

## 3. Results and Discussion

### 3.1. Synthesis of Two Types of Environmentally Friendly Absorbents 

**Figure 1:**
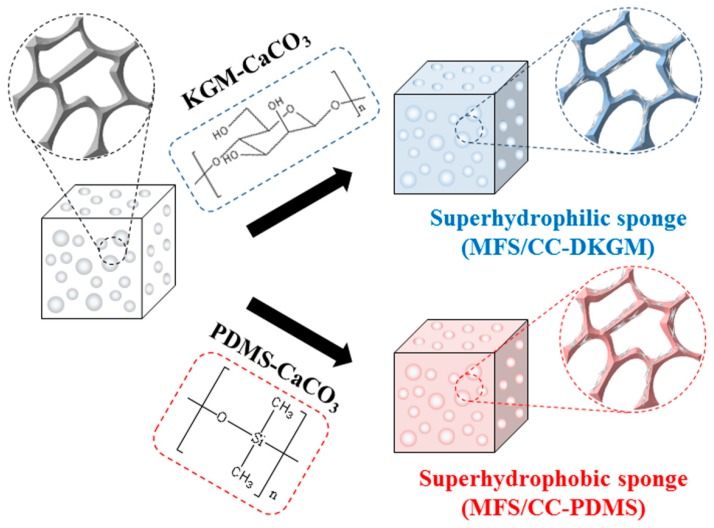
Schematic illustration of the one-step synthesis of environmentally friendly superhydrophilic (MFS/CC-DKGM) and superhydrophobic (MFS/CC-PDMS).

Figure 1 shows a schematic illustration of the synthesis of two types of environmentally friendly absorbents (i.e., superhydrophilic and superhydrophobic absorbents). A melamine formaldehyde sponge (MFS) had been used as a base material for various types of applications, due to its easy surface modification and excellent durability [16,18,23,26,27,28]. The superhydrophilic absorbent (MFS/CC-DKGM) was prepared by one-step coating of an MFS with a mixture of calcium carbonate (CC) rods and Konjac glucomannan (KGM). The superhydrophobic absorbent (MFS/CC-PDMS) was also prepared by one-step coating of an MFS with a mixture of CC rods and polydimethylsiloxane (PDMS). During the CC-DKGM- and CC-PDMS-coating process of the MFS, the KGM was deacetylated into deacetylate KGM (DKGM), and DKGM and PDMS were cross-linked, which resulted in fixation of the CC rods within the three-dimensional cross-linked network. The mechanical strength of each sponge can be enhanced by cross-linking. KGM is a food additive and a water-soluble polysaccharide, and, thus, is a biopolymer and biodegradable. PDMS is inert and nontoxic. CC is the main component of pearls and the shells of marine organisms and is used medicinally, as a calcium supplement or antacid. The abovementioned materials were selected because of their environmentally friendly and nontoxic characteristics and their abilities to improve the mechanical strength after cross-linking.

### 3.2. Surface Morphology of MFS/CC-DKGM and MFS/CC-PDMS 

**Figure 2:**
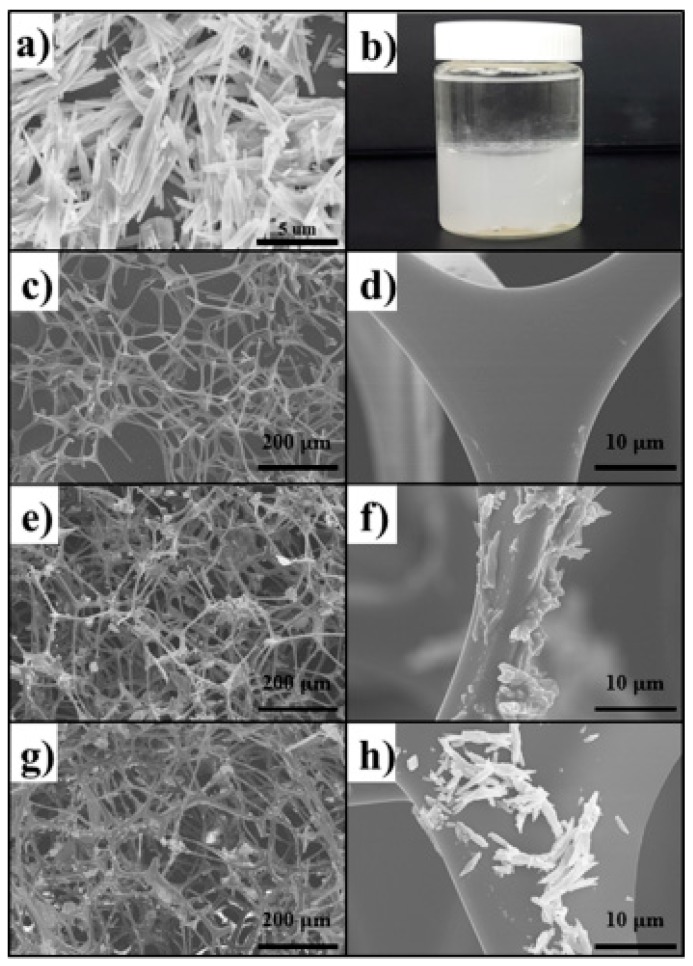
(**a**) A SEM image of the CC rods. (**b**) Image of CC rods dispersed in a hexane/water mixture. SEM images of (**c**,**d**) MFS, (**e**,**f**) MFS/CC-DKGM, and (**g**,**h**) MFS/CC-PDMS.

Figure 2a shows the scanning electron microscopy (SEM) image of CC rods with an average size of 12 µm. CC dispersed only in the water layer of the hexane/water mixture, demonstrating its hydrophilicity (Figure 2b). Figure 2c–h shows SEM images of the MFS before and after coating with CC-DKGM and CC-PDMS. The MFS showed a smooth surface morphology and a 3D interconnected network skeleton (Figure 2c,d). Conversely, after CC-DKGM coating, the surface morphology analysis showed CC rods on the MFS surface (Figure 2e,f), proving that the MFS surface was coated with CC-DKGM. The CC rods were tightly fixed within a three-dimensional cross-linked DKGM network. The acetyl groups on KGM were replaced by hydroxyl groups after heating, thereby increasing the hydrogen bonding between the KGM chains [29,30]. During this reaction, the CC rods were naturally surrounded and fixed by a three-dimensional cross-linked DKGM network. This analogous surface morphology was also observed in the MFS/CC-PDMS, indicating successful coating of the MFS surface with CC-PDMS (Figure 2g,h). CC was used to induce a rough structure on the smooth surface of the MFS and to achieve a higher or lower water-contact angle (WCA) for the MFS/CC-PDMS or MFS/CC-DKGM, respectively. 

### 3.3. Characterization of MFS/CC-DKGM and MFS/CC-PDMS 

**Figure 3:**
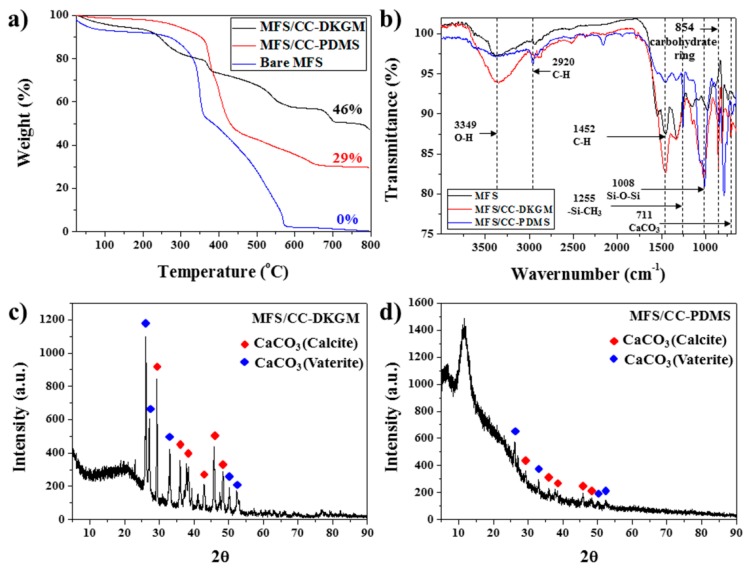
(**a**) TGA data for the (black line) MFS/CC-DKGM and (red line) MFS/CC-PDMS. (**b**) FT-IR data for the (black line) MFS, (red line) MFS/CC-DKGM, and (blue line) MFS/CC-PDMS. XRD data for the (**c**) MFS/CC-DKGM and (**d**) MFS/CC-PDMS.

The thermogravimetric analysis (TGA) data revealed that the CC portions accounted for 46% and 28% of the MFS/CC-DKGM and MFS/CC-PDMS, respectively, compared to that of the MFS (Figure 3a). The CC rods were loaded at much higher concentrations on the MFS/CC-DKGM than on the MFS/CC-PDMS, because KGM had a lower viscosity than PDMS. Fourier transform-infrared (FT-IR) spectroscopy was performed to confirm formation of the MFS/CC-DKGM and MFS/CC-PDMS (Figure 3b). The peaks at 3349 cm^−1^ and 854 cm^−1^ originated from the -OHand carbohydrate ring-stretching vibrations, respectively, which are the characteristic groups in DKGM, as shown by the red line (in Figure 3b) [31]. This finding proves that the MFS was coated with DKGM (black and red lines). The new absorption peaks at 2920 cm^−1^ (aliphatic CH) and 1255 cm^−1^ (Si-CH_3_) corroborated coating of the MFS with PDMS (black and blue lines). The new absorption peak at 711 cm^−1^ (CaCO_3_) indicated incorporation of the CC rods into the MFS/CC-DKGM and MFS/CC-PDMS. X-ray diffraction (XRD) analysis was performed to further confirm the formation of MFS/CC-DKGM and MFS/CC-PDMS. Both MFS/CC-DKGM and MFS/CC-PDMS exhibited typical XRD patterns of the CC (CaCO_3_) structure (JCPDS 001-0482), which indicated that the CC rods were the mixture of calcite and vaterite phases (Figure 3c,d). MFS only showed amorphous phase at the range of 10^°^–25^°^ (2). The SEM, FT-IR, and XRD data suggested that the MFS was successfully coated with layers of CC-DKGM and CC-PDMS by applying a one-step coating mixture of CC and KGM (or PDMS).

### 3.4. Wettability Characteristics of MFS/CC-DKGM and MFS/CC-PDMS 

**Figure 4:**
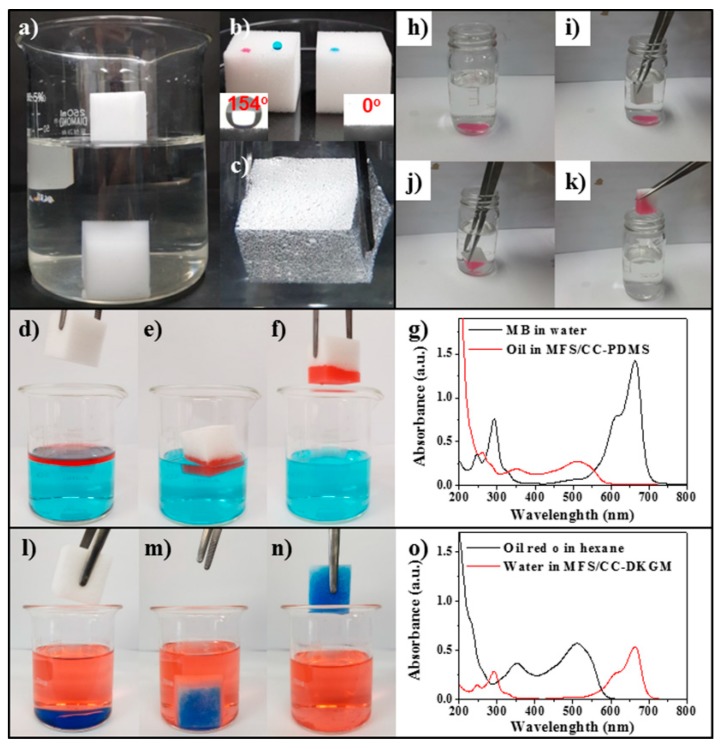
(**a**) Image showing the MFS/CC-DKGM (submerged under water, bottom of beaker) and MFS/CC-PDMS (floating on top of the water, top of beaker). (**b**) The image on the right side of the larger image on the left shows oil and water droplets on the MFS/CC-PDMS and (right) water droplets on the MFS/CC-DKGM. The insets show the WCAs on the (left) MFS/CC-PDMS and (right) MFS/CC-DKGM. (**c**) Image of the MFS/CC-PDMS immersed in water by an external force. (**d**–**f**) Images showing spontaneous oil absorption of the MFS/CC-PDMS in a hexane/water mixture. (**g**) UV-vis absorption spectrum (red line) of oil red O solution obtaining from the MFS/CC-PDMS. (black line) For comparison, UV-vis absorption spectrum of MB solution was provided. (**h**–**k**) Images showing the chloroform absorption and separation processes from the water/chloroform mixture using the MFS/CC-PDMS. (**l**–**n**) Images showing the water absorption and separation processes from the hexane/water mixture, using the MFS/CC-DKGM. (**o**) The red line represents the UV-vis absorption spectrum of MB solution obtained from the MFS/CC-DKGM. For comparison, the black line represents UV-vis absorption spectrum of oil red O solution was provided.

Several tests were performed to investigate the characteristics of the MFS/CC-DKGM and MFS/CC-PDMS. The MFS/CC-DKGM and MFS/CC-PDMS showed significantly different wetting properties. When these absorbents were added to water, the MFS/CC-DKGM sank to the bottom of the water due to its hydrophilicity, but the MFS/CC-PDMS floated on top of the water due to its hydrophobicity (Figure 4a). A water droplet (blue-colored with MB dye) formed a spherical shape on the MFS/CC-PDMS, whereas a hexane droplet (light oil, red-colored with oil red O dye) quickly absorbed onto the MFS/CC-PDMS (Figure 4b, left). Conversely, a water droplet rapidly absorbed on the MFS/CC-DKGM (Figure 4b, light). The water-contact angles (WCAs) of the MFS/CC-DKGM and MFS/CC-PDMS were 0° and 154°, indicating their superhydrophilicity and superhydrophobicity, respectively (see insets of Figure 4b). When the MFS/CC-PDMS was immersed in water, a silver mirror-like phenomenon was observed on the surface of the MFS/CC-PDMS that corresponded to Cassie-Baxter nonwetting behavior due to the presence of an air layer between the surrounding water and the trapped air in the MFS/CC-PDMS (Figure 4c). This result further confirmed the hydrophobicity and oleophilicity of the MFS/CC-PDMS. When the MFS/CC-PDMS was added to a hexane-and-water mixture, the MFS/CC-PDMS quickly and selectively absorbed hexane on the water (Figure 4d–f). The presence of water residue (blue-colored with MB dye), in the collected hexane, was investigated by detection of MB. The UV-vis absorption data revealed that the MFS/CC-PDMS absorbed only hexane (Figure 4g). The MFS/CC-PDMS selectively absorbed chloroform (heavy oil, red-colored with oil red O dye) under the water when the MFS/CC-PDMS was immersed into an oil/water mixture (Figure 4h–k). After absorption of oils, no visible water was observed in the collected chloroform sample. These results indicate that the MFS/CC-PDMS is able to selectively absorb light and heavy oils from oil/water mixtures. The selective absorption characteristic of the MFS/CC-DKGM was also evaluated. When the MFS/CC-DKGM was immersed into an oil/water mixture, the MFS/CC-DKGM selectively absorbed water under hexane (red-colored with oil red O dye) (Figure 4l–n). After the absorption of water, no visible hexane was observed in the collected water. The presence of oil residue (red-colored with oil red O dye) in the collected water was also investigated by detection of oil red O. The UV-vis absorption data revealed that the MFS/CC-DKGM absorbed only water (Figure 4o). These data proved that the MFS/CC-DKGM and MFS/CC-PDMS could selectively absorb water and oil from oil/water mixtures, respectively.

### 3.5. Oil and Water Absorption Performances of MFS/CC-DKGM and MFS/CC-PDMS 

**Figure 5:**
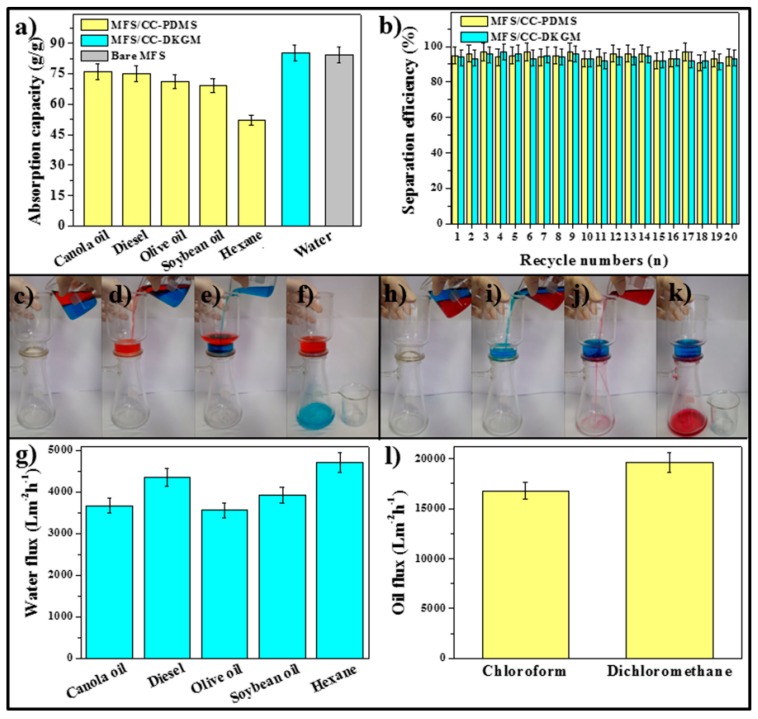
(**a**) Absorption capacities of the MFS/CC-PDMS and MFS/CC-DKGM for various types of oils and water, respectively. (**b**) Reusability tests for hexane and water, with more than 20 consecutive cycles using the MFS/CC-PDMS and MFS/CC-DKGM, respectively. Images showing the oil/water separation process using (**c**–**f**) MFS/CC-DKGM and (h–k) MFS/CC-PDMS prewetted with (**c**–**f**) water and (**h**–**k**) oil, respectively. Separation capacities (flux) for (**g**) water and (**l**) oil using the (**g**) MFS/CC-DKGM and (**l**) MFS/CC-PDMS, respectively.

To investigate the oil and water absorption performances from oil/water mixtures, the oil and water absorption tests from the oil/water mixtures were performed by placing a piece of MFS/CC-PDMS (8 cm^3^) and MFS/CC-DKGM (8 cm^3^) on a mixture of various oils. The MFS/CC-PDMS showed excellent oil-absorption capacities, which reached 52 g/g–76 g/g from various oil/water mixtures over 5 species (Figure 5a). The lowest absorption capacity was observed for hexane because of its volatile characteristic. The MFS/CC-PDMS also exhibited excellent recyclability, with high-separation efficiency (91%–97%) for hexane up to 20 times (Figure 5b). The MFS/CC-DKGM exhibited the best water-absorption capacity of 86 g/g and maintained the high-separation efficiency (92%–97%) up to 20 times (Figure 5a,b). To investigate the feasibility of their use as a filter, oil/water separation tests of MFS/CC-PDMS (8 cm^3^) and MFS/CC-DKGM (8 cm^3^) were performed using the filter process. After the MFS/CC-DKGM was prewetted with water; only water (colored blue with MB) rapidly penetrated the MFS/CC-DKGM as soon as the hexane (colored red with oil red O) and water mixture was poured into a funnel equipped with the MFS/CC-DKGM filter (Figure 5c–f). Various oils with different viscosities, including diesel fuel (μ = 2.0 cP), hexane (viscosity, μ = 0.29 cP), olive oil (μ = 107.5 cP), canola oil (μ = 83.0 cP), and soybean oil (μ = 60.0 cP), were used to investigate the separation capacity (flux) of the MFS/CC-DKGM filter. The MFS/CC-DKGM filter exhibited excellent flux performances, which reached 4,702 L/m^2^ h – 3,562 L/m^2^ h (Figure 5g). No visible oil was observed in the collected water. After the MFS/CC-PDMS filter was prewetted with oil, only chloroform or dichloromethane (colored red with oil red O) was able to selectively pass through the MFS/CC-PDMS filter when the oil/water mixture was poured into a funnel (Figure 5h–k). No visible water was observed in the collected oils. The fluxes of the MFS/CC-PDMS filter were 16,793 L/m^2^ h for chloroform and 19,591 L/m^2^ h for dichloromethane, respectively (Figure 5l). Water and oil layers formed at the surfaces of the MFS/CC-DKGM and MFS/CC-PDMS and blocked the passage of oil and water, respectively, which resulted in the passage of water and oil alone. For comparison, water absorption and filtration tests were performed using the MFS. The absorption capacity of the MFS was of 84 g/g, which was comparable to that of MFS/CC-DKGM (Figure 5a). However, the MFS was unable to filter water when it was used as a filter. The MFS absorbed water as well as oil because the MFS was less hydrophilic than the MFS/CC-DKGM, which restricted water penetration. An overall performance comparison of different oil/water separators is summarized in Table 1 [32,33,34,35,36,37,38,39], which clearly shows that MFS/CC-DKGM and MFS/CC-PDMS have outstanding performance, considering absorption capacity and flux. The flux of MFS/CC-PDMS was comparable to the best ever reported in the literature, while the absorption capacity of MFS/CC-DKGM was the best and incomparable to those of the others reported in the literature (Table 1). These results demonstrate that MFS/CC-DKGM and MFS/CC-PDMS can be used as an absorbent and filter for oil/water separation.

### 3.6. The Chemical, Thermal, and Mechanical Stabilities of MFS/CC-DKGM and MFS/CC-PDMS 

**Figure 6:**
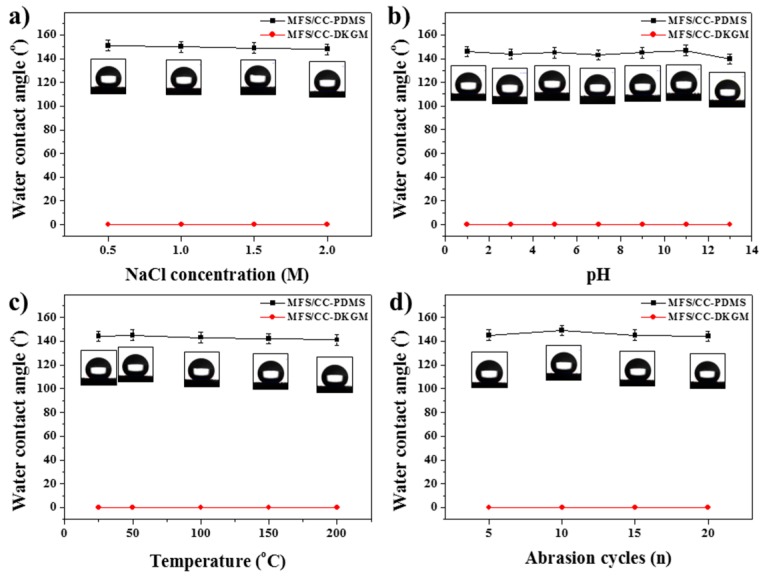
WCA data for the MFS/CC-DKGM and MFS/CC-PDMS under various stimuli: (**a**) salt, (**b**) pH, (**c**) heat, and (**d**) mechanical abrasion treatments.

The chemical, thermal, and mechanical stabilities of the MFS/CC-PDMS and MFS/CC-DKGM were tested by measuring variations in the WCA after exposing the sponges to different conditions for 6 h. Under various chemical conditions (i.e., in the presence of salt, NaCl and different pH values), the WCAs of the MFS/CC-PDMS decreased slightly but remained above 140°, and the MFS/CC-DKGM maintained its superhydrophilicity, which suggests that the hydrophobicity and superhydrophilicity of the MFS/CC-PDMS and MFS/CC-DKGM, respectively, were robust against corrosive environments (Figure 6a,b). The thermal stability of the MFS/CC-PDMS and MFS/CC-DKGM were also tested by subjecting the sponges to a high temperature of 200 °C for 5 h; the results revealed that the WCAs of the MFS/CC-PDMS decreased slightly but remained above 141°, and MFS/CC-DKGM maintained the superhydrophilicity (Figure 6c). For the mechanical abrasion test, sandpaper with a load of 500 g was brought directly into contact with the MFS/CC-PDMS or MFS/CC-DKGM (8 cm^3^). The sample was dragged along a 0.3 m distance with a pull speed of 0.1 m/s with horizontal and vertical directional movements. After the mechanical abrasion test, the WCAs of the MFS/CC-PDMS decreased slightly but remained above 142°, and MFS/CC-DKGM maintained the superhydrophilicity for 20 abrasion cycles, indicating a relatively good resistance to mechanical abrasion (Figure 6d). Abovementioned results indicate that the MFS/CC-DKGM and MFS/CC-PDMS maintained their wettability characteristics relatively well after the chemical, thermal, and mechanical stability tests.

### 3.7. The Possibility of Leakage of CC Rods from MFS/CC-DKGM and MFS/CC-PDMS after Use

**Figure 7:**
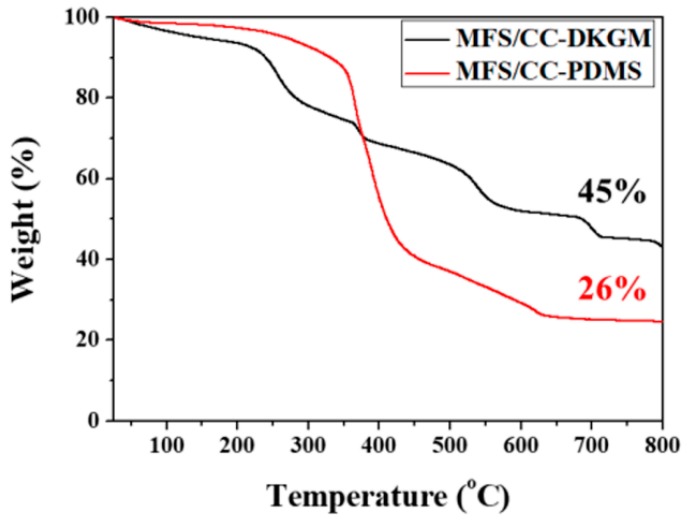
TGA data for the MFS/CC-DKGM and MFS/CC-PDMS after the oil/water separation test.

To investigate the possibility of leakage of CC rods, the CC-rod contents on the MFS/CC-DKGM and MFS/CC-PDMS were compared before and after oil/water separation. The CC portions accounted for 45% and 26% of the MFS/CC-DKGM and MFS/CC-PDMS after oil/water separation up to 20 cycles, which were slightly decreased compared to the CC portions of 46% and 28% of the MFS/CC-DKGM and MFS/CC-PDMS before oil/water separation, respectively (Figure 7). The TGA data revealed no remarkable CC-rod content changes before and after oil/water separation when each sponge was used for this process. CC is the main component of shells of marine organisms. KGM is a food additive, which is a biopolymer and is biodegradable. PDMS is inert and nontoxic. Since the abovementioned materials are part of nature, biodegradable, or nontoxic, they do not cause any issues even if they leak into the natural environment. We recently reported an anti-overturn Janus sponge and potential amphiprotic sponge for simultaneous pollutant remediation and oil/water separation [18,40]. Both sponges possessed amphiprotic characteristics, which enabled these sponges to separate oil/water mixture, including emulsion and purify pollutants simultaneously. We expect that the MFS/CC-DKGM can be used for simultaneous oil/water separation and pollutant purification because the MFS/CC-DKGM possessed various functional groups to remove organic pollutants and selective wettability to separate oil/water mixture.

## 4. Conclusions

In conclusion, we demonstrated the one-step synthesis of environmentally friendly superhydrophilic and superhydrophobic sponges for oil/water separation, which has not yet been reported. The MFS/CC-DKGM (or MFS/CC-PDMS) was synthesized by one-step coating of an MFS with a mixture of CC rods and KGM (or PDMS). The MFS/CC-PDMS showed excellent absorption capacity, which reached 52 g/g–76 g/g following immersion into various types of oil/water mixtures. Furthermore, after the MFS/CC-DKGM and MFS/CC-PDMS were prewetted with water and oil, respectively, only water and oil quickly penetrated the respective sponges as soon as the oil/water mixture was poured into a funnel equipped with the MFS/CC-DKGM and MFS/CC-PDMS filters. The MFS/CC-DKGM and MFS/CC-PDMS filters exhibited excellent water and oil flux performances, which reached 4,702 L/m^2^h and 19,591 L/m^2^h, respectively. The flux of MFS/CC-PDMS was comparable to the best-ever reported in literature, while the absorption capacity of MFS/CC-DKGM was the best and incomparable to those of the others reported in the literature. The MFS/CC-DKGM and MFS/CC-PDMS maintained their wettability characteristics relatively well after the chemical, thermal, and mechanical stability tests. 

## Figures and Tables

**Table 1:** Performance comparison of different oil/water separators.

Materials	Types of Liquid	Absorption Capacity (g/g)	Flux (L/m^−2^h^−1^)	Ref.
Hydrolyzed luffa sponge	Water	75	-	[32]
MFS/Fe_3_O_4_/CS-PFNA	Water	65.7	-	[33]
PUS/CNT/Pdop/ODA	n-Hexane	34.9	-	[34]
PUS/Graphene	n-Hexane	29.5	-	[35]
Cu mesh/Cement	Water/n-Hexane	-	5000 (Water)	[36]
TiO_2_-PVDF membrane	Water/n-Hexane	-	785 (Water)	[37]
Cellulose fiber/LDH	Water/Chloroform	-	4968 (Chloroform)	[38]
SS mesh/Potato residue	Water/Chloroform	-	32400 (Chloroform)	[39]
MFS/CC-DKGM	Water Water/n-Hexane	85.2 -	- 4702 (Water)	This work
MFS/CC-PDMS	n-Hexane Water/Chloroform	52 -	- 16793 (Chloroform)	This work
